# A Critical Examination of the Certified Community Behavioral Health Clinic Model: Provider Perceptions and Themes

**DOI:** 10.1111/1475-6773.70041

**Published:** 2025-09-07

**Authors:** Tugba Olgac, Emma McCann, Michelle Riske‐Morris, David L. Hussey

**Affiliations:** ^1^ Begun Center for Violence Prevention Research and Education, Jack, Joseph and Morton Mandel School of Applied Social Sciences Case Western Reserve University Cleveland Ohio USA

## Abstract

**Objective:**

To explore the experiences of providers from two community behavioral health agencies involved in the implementation of Certified Community Behavioral Health Clinics (CCBHCs).

**Study Setting and Design:**

This qualitative study was conducted as part of a larger evaluation of CCBHC implementation outcomes in two community‐based behavioral health agencies. Ninety‐one participants, including case managers, counselors, care coordinators, and leadership teams from both agencies, participated in focus group discussions to share their experiences regarding the implementation of the CCBHC model within their organizations.

**Data Sources and Analytic Sample:**

Three rounds of focus group discussions were held between 2021 and 2023. A total of 24 focus groups were audio‐recorded and transcribed by one of the researchers. Qualitative data was analyzed by two researchers using the systematic text condensation method.

**Principal Findings:**

Six themes emerged from the focus groups reflecting both positive impacts and implementation challenges. Providers reported the implementation of CCBHCs improved service accessibility and effective care coordination; however, staff noted difficulties connecting clients with essential community resources, including housing and transportation. Both agencies underwent significant organizational transformation, although communication strategies varied by agency size. Finally, providers observed improved communication, client benefits (e.g., reduced hospitalizations), and positive organizational change. Despite these successes, agencies expressed significant concerns about long‐term program viability due to reliance on temporary grant funding.

**Conclusion:**

The CCBHC model of integrated care has expanded significantly in recent years. Most participants reported a positive cultural shift within their agencies following CCBHC implementation. However, limited community resources continue to restrict agencies' ability to address clients' basic needs. Since the CCBHC model was implemented through temporary grant funding, sustainability remains a concern. Both issues underscore the need for policies that increase the availability of community resources and ensure the long‐term viability of CCBHCs.

**Summary**

- What is known on this topic
  ○ Certified Community Behavioral Health Clinic (CCBHC) is a model of integrated care that aims to increase access to high‐quality services.
  ○ The emerging literature suggests that CCBHCs are related to reduced emergency department visits and hospitalizations.
  ○ There is a limited knowledge of provider experiences with CCBHC implementation.
- What this study adds
  ○ Providers reported multiple positive outcomes, including enhanced service accessibility, improved communication and collaboration, effective care coordination, observable client benefits, and organizational transformation.
  ○ Providers also encountered difficulties connecting clients with essential community resources such as housing and transportation.
  ○ Providers expressed concerns about the long‐term viability of CCBHCs and employment of care coordinators due to their reliance on temporary grant funding.

## 1 Introduction

Individuals with mental illness and/or substance use disorders (SUDs) have long faced difficulties with accessing and maintaining the care they need, including medical care [1]. Access to medical care is crucial because those with severe mental illness and/or SUDs are at increased risk for severe medical problems. For example, a meta‐analytic study found that individuals with schizophrenia were at increased risk for cardiovascular diseases including coronary heart disease, stroke, and congestive heart failure [2]. Similarly, SUDs are associated with a number of medical conditions such as cancer, sexually transmitted diseases, and cardiovascular diseases as well as mental health problems [3]. These co‐occurring and often chronic conditions require coordinated, long‐term care. Unfortunately, healthcare in the United States remains largely compartmentalized, despite efforts toward integration.

One federal initiative implemented to promote integrated care and increase access to services was the formation of Certified Community Behavioral Health Clinics (CCBHCs). CCBHCs are funded through the U.S. Department of Health and Human Services (HHS) with the goal of increasing access to timely, high‐quality, evidence‐based behavioral health services regardless of a client's ability to afford them [4]. CCBHCs are certified based on six key criteria: staffing, availability and accessibility of services, care coordination, scope of services, quality and reporting, and organizational authority [4, 5]. They are expected to expand their staff and services to ensure timely access and meet clients' cultural, linguistic, and behavioral healthcare needs [4, 5].

CCBHCs provide evidence‐based practices (EBPs) in behavioral health, collaborate with primary care providers to address co‐occurring medical conditions, and work with designated collaborating organizations (DCOs) to offer mobile crisis services and other community‐based services that address social needs [4, 5, 6]. Currently, there are 495 CCBHCs operating across 46 states, Washington D.C., and Puerto Rico, serving an estimated 3 million people [7]. While there is a growing body of gray literature reporting on the implementation and impact of CCBHCs, peer‐reviewed research on this topic remains limited. Evaluations of the CCBHC demonstration program and accompanying CCBHC grants have suggested that CCBHCs are successful in achieving their goals [4, 6, 8]. CCBHC demonstration sites reported hiring more staff to meet client needs [4, 6, 8, 9], improving access through same‐day services and screening most clients within 10 days, expanding services including mobile crisis management [4, 9] and community behavioral health programs [4, 8, 9], integrating primary care [4, 8], addressing client social needs [9], providing more EBPs and reducing client involvement with law enforcement and emergency services [4, 8].

Nevertheless, peer‐reviewed literature on CCBHCs is scarce, and very few addressed the impact of CCBHCs. In their analysis of Medicaid claims data from 2015 to 2019 across three states, Brown et al. (2023) found that CCBHCs were associated with lower utilization of emergency departments (EDs) in Pennsylvania and Oklahoma, but not in Missouri [10]. Analyses also suggested some evidence for the reduced all‐cause hospitalizations in Pennsylvania and Oklahoma [10]. Another study comparing non‐Medicaid CCBHC clients with severe mental illness and non‐CCBHC clients in New York found an increase in mental health service utilization and reductions in ED visits and non‐psychiatric hospitalizations for the CCBHC clients [11]. Additionally, a mixed‐methods study explored client and provider experiences with the CCBHC, finding that clients were satisfied with the primary care services offered at the clinic and the collaboration between their providers. In contrast, providers expressed less satisfaction with the level of collaboration and integration at the clinic [12].

The peer‐reviewed CCBHC literature has primarily analyzed claims data to evaluate large‐scale implementation outcomes. While quantitative research and evaluations indicate CCBHC effectiveness in reducing ED utilization and improving access, the perspectives of providers implementing these complex organizational changes remain largely unexplored. Understanding provider experiences is crucial for several reasons: implementation success depends heavily on provider buy‐in; provider insights can identify practical barriers not captured in quantitative data; and lessons learned from early implementers can inform future CCBHC implementation efforts. This study addresses this knowledge gap by examining the lived experiences of providers during CCBHC implementation.

## 2 Methods

Focus groups were conducted as part of local evaluations with staff from two behavioral health agencies in the Midwest, both of which received two‐year CCBHC grants in 2021. Potential participants were identified, and focus groups were scheduled with assistance from agency staff members. Three rounds of focus group discussions were completed with each agency, once every 6 months, starting from the end of the first year of the grant in 2021 through 2023. After each round of focus groups, the focus group guides were reviewed, and improvements, such as new probing questions, were added. All focus groups covered the topics of access to services, the role of care coordinators within the organization, client benefits, continuity of care, and lessons learned. Additionally, focus groups with administration and leadership included questions about CCBHC planning and information regarding any changes made during implementation. As part of two local evaluations, the focus group protocols and interview guides were reviewed by the institutional review board and deemed exempt.

Originally, 26 focus groups were conducted. However, two focus groups were excluded from the analysis, since they lasted less than 15 min and did not provide sufficient information to inform our study. Many of the participants from these groups were also present in other focus groups, allowing them to share their insights. This resulted in 24 focus groups for analysis (14 from Agency A and 10 from Agency B), comprising 91 unique participants, with many participants present at multiple focus groups across the three time points. Focus groups lasted 20 to 61 min, with an average length of 37 min, and were audio‐recorded. Initial transcripts were generated through Otter.ai, then reviewed and cleaned by one of the interviewers to ensure accuracy.

## 3 Analysis Strategy

Two researchers created a preliminary codebook based upon the thematic analysis of the focus groups and subsequently revised the codebook after reviewing all 24 transcripts together. Both researchers then coded each transcript through NVivo using the revised codes. After the researchers reached agreement on the coded transcripts, the qualitative data were analyzed and re‐analyzed by the same researchers using the Systematic Text Condensation method [13]. The researchers read and re‐read the quotes within each code, pulling preliminary and subsequently emerging themes from the broader context of the codes and grouping them together into discrete meaning units. The contents of these meaning units were extracted, sorted, and condensed into one document to provide detailed descriptions of each theme. Analytical rigor was maintained through independent coding of all 24 transcripts using the agreed‐upon codebook and holding regular consensus meetings to discuss and resolve any coding disagreements. Researchers continuously compared data with existing codes, categories, and ideas using an iterative approach.

Following each wave of data collection, the research team reviewed preliminary themes and assessed whether new information emerged. Saturation was considered achieved when no new themes or significant variations in existing themes emerged from subsequent focus groups, and when the data provided sufficient depth to understand the range of experiences across both agencies. The three‐round design allowed for longitudinal tracking of implementation experiences, with the final round capturing perspectives on CCBHC sustainability concerns. The findings are paraphrased, with representative direct quotes from participants presented in a table, organized by corresponding themes.

## 4 Results

Throughout the two‐year CCBHC grant period, Agency A served a total of 203 clients (189 adults, 14 youth), while Agency B served 525 clients (363 adults, 162 youth). The majority of the clients served at Agency A (82%) were White, and 9% were African American, representative of the county population. Agency B served a higher percentage of African American clients (42%) relative to the county population, with White clients comprising 48% of the client population. Participants in this study included direct service providers and leadership teams (*n* = 91). A breakdown of the number of participants in each provider team is shown in Table 1. Overall, they had a mean of 4.14 years of experience (SD = 5.39), ranging from 0.08 to 25 years. Participants from Agency A had a higher mean of years of experience (M = 4.52, SD = 5.91) than participants from Agency B (M = 3.37, SD = 4.16).

**TABLE 1:** Participant characteristics.

Role	Agency A participants: *N* (%)	Agency B participants: *N* (%)	Total *N* (%)
Leadership and administration	12 (19.7)	12 (40.0)	24 (26.4)
Case management	17 (27.9)	2 (6.7)	19 (20.9)
Care coordination	5 (8.2)	4 (13.3)	9 (9.9)
Counselor/therapist	6 (9.8)	3 (10.0)	9 (9.9)
Medical	6 (9.8)	1 (3.3)	7 (7.7)
SUD^a^ assessment/treatment	0 (0)	6 (20.0)	6 (6.6)
Supportive employment	5 (8.2)	0 (0)	5 (5.5)
Supervisors	5 (8.2)	2 (6.7)	7 (7.7)
Other	5 (8.2)	0 (0)	5 (5.5)
Total	61 (67.0)	30 (33.0)	91 (100)

^a^ Substance use disorder.

Analyses revealed six themes: access to services, care coordination, communication and collaboration, client benefits, organizational change, and sustainability. These themes represent the primary areas of positive impact and challenges experienced by both agencies during CCBHC implementation. Representative direct quotes from participants for each corresponding theme are presented in Table 2.

**TABLE 2:** Themes and example quotes.

Theme	Example quotes
Access to Services	“We've been able to help a few clients specifically with some really significant and chronic physical health issues, able to manage those better and get those needs met. Because a lot of it for them had been that they were going to doctors before, who weren't considering their mental illness and just weren't explaining things or making prescribing decisions that met the needs that were coming from those significant illnesses, those significant mental illnesses.” [Agency A] “Transportation is a huge barrier specific to this community. You know, the public transportation options for people are now limited even more due to the [county transit agency] being understaffed … There's just not a lot of resources that are reliable and timely. So, that I think is a huge barrier to people getting the services that they need.” [Agency A] “I think before we started this process, it was 6 weeks for an adult to get in, right. Now, I was just looking at the schedule, and I can get someone in next week for an assessment.” [Agency B] “I think it's not unique to the grant, but I think the healthcare world has been shaken up. And there's challenges with recruitment, there's some turnover. And I think that leads to some people disengaging from services.” [Agency B]
Care Coordination	“We also saw not just different levels of care within our own organization, but supporting our folks getting access to other levels of care within other organizations.” [Agency A] “One of the things that leads to most of our burnout is just feeling like we don't have the capacity to meet all the needs of the clients that we might have. So, I've definitely, particularly case managers, I've had them say, like how beneficial it's been to have a care coordinator and a partner that they can, you know, tag in, or get some added support from.” [Agency A] “I think that there's just lots of confusion on what case management work or role is and what CCBHC^a^ role is for clients, you know. And then new workers like myself as well, until, again, we get a ton of support from, like, our team members, and our supervisors in clarifying that…it's just very similar.” [Agency A] “Care coordination, again, also like helping people get to the right service at the right time, it's more preventive, like helping reduce the likelihood of a crisis. They deal with people in crisis, too. But I kind of think about those that act in care coordination as sort of a preventive effort.” [Agency B] “So, [a care coordinator] sounds like a case manager with a new name. Now that we canceled case management services, we have peer coordination, which we really do ourselves.” [Agency B]
Communication and Collaboration	“I've worked with a couple clients that are diabetic and homeless and don't have access to be able to maintain their diabetic supplies, dispose of their needles, keep their insulin refrigerated, you know, things like that. And we've been able to work with our pharmacy and a couple other local pharmacies … and a lot of them were very open to storing the medication there if we would bring them in weekly, and then the client could give themselves their injection.” [Agency A] “We used to not even know who the doctors were. Now, the doctors come over and say, ‘hey, I want to consult about this. I want to talk about this client,’ and we can do it vice versa. And I'll tell you, that's fantastic.” [Agency B] “I look at it as like, there's the pharmacy, and there's urgent care. And I think like, we communicate with each other, but we're very within our own department sometimes.” [Agency B]
Client Benefits	“We saw better outcomes. And we also saw better client reports of their own wellness, which was key. So, we had clients themselves self‐reporting that they felt better, that they felt like they had options that were available to them. So, you know, those kinds of things help in the stability of our individuals long term.” [Agency A] “When you look at each client, you can tell, you know, how well they've been doing with, for example, we have a couple of clients who are always in and out of the hospital. We still have one or two, but overall, you know, we were able to help reduce their hospitalizations, their ED visits, you know, for their mental health.” [Agency B]
Organizational Change	“CCBHC is not a department unto itself. CCBHC is the agency. Because I think another thing that I didn't see coming was the number of staff that were going to touch it … this is a transformational organizational project, not a grant that lives kind of in its own little area.” [Agency A] “I think that your team is only as strong as your leaders … our leaders put a tremendous amount of faith and trust in us and gave us the opportunity to make this what it was. They let us grow, they let us advocate, which in this field, you don't often get an opportunity to do that.” [Agency A] “There's more of this comprehensive wraparound kind of flavor with the services that we provide … I think the whole agency has started to understand the benefit of treating the whole client, and not just that one piece … I see a whole difference to this whole agency and the way we treat a person.” [Agency B] “There's just more training, we're able to really set a culture of ongoing learning … and then also sends the message that we value you as a clinician to be able to support people through these trainings.” [Agency B]
Sustainability	“Currently, that's [care coordination is] just not a structure that is endorsed within the [state] landscape. You're seeing the governor's office start to take that on with [state care coordination program for youth] and putting significant dollars in investment. I would like to see that same enthusiasm for our adult patients, because, regrettably, most of those kids that are in [state care coordination program for youth] are eventually going to age into the adult system.” [Agency A] “My concern since day one has making sure that they're [care coordinators are] viable. Because they have very limited billing. So, making sure that we have funds to support the role because they're not producing revenue for us, in the true sense of, you know, this service gets billed out directly attached to them. So, I think that's the biggest thing that I have had concerns with it throughout, is being able to make sure that we have a financial process in place to support them.” [Agency B]

^a^ CCBHC, Certified Community Behavioral Health Clinic.

### 4.1 Access to Services

Focus group discussions regarding access to services identified four key categories: challenges with access to community resources including transportation and housing, staffing shortages, and expanded access to services within the clinic. Concerns about barriers to transportation and housing resources in the community were raised by both agencies. At Agency A, care coordinators were instrumental in navigating transportation resources, yet challenges persisted due to an understaffed county transportation line and structural issues, such as a lack of wheelchair accessibility. Care coordinators at Agency B reported employing various strategies to assist clients with transportation needs, including contacting insurance providers, reimbursing rides provided by clients' relatives or friends, or arranging commercial rides. However, these strategies were not helpful for some clients. For example, commercial rides presented challenges for clients with communication difficulties or complex needs related to their mental health conditions, and not all clients had personal connections who could provide transportation.

Both agencies reported challenges with housing resources. Agency A care coordinators highlighted the scarcity of housing in the county, particularly for individuals with disabilities and low incomes. Despite these difficulties, they achieved some success in helping clients obtain housing vouchers and secure group home placements. Agency B care coordinators emphasized the challenges of finding housing in high‐demand areas, noting that while some resources existed, they were often in less desirable locations.

Additionally, both agencies reported struggling with a shortage of case managers. Care coordinators had to assume case management duties, and at Agency B, the counseling team also handled case management responsibilities at times to ensure clients' basic needs were met before counseling could proceed. This shortage was exacerbated by high turnover and inadequate compensation, leading to case managers frequently transitioning into other roles.

Despite these challenges, both agencies were able to expand service access through CCBHC grant funding. The funding enabled new services for specific populations, such as veterans and individuals with severe mental illness, and integrated primary care within their facilities. Agency A staff noted that they made significant progress toward holistic care by enhancing the integration of behavioral and physical health services, which led to reduced hospitalizations and fewer legal issues among clients. Similarly, staff at Agency B reported that access to primary care was improved by direct scheduling of services and an increased number of primary care staff. The addition of care coordination and Assertive Community Treatment (ACT) services increased client engagement and connected high‐need clients to necessary services. At both agencies, care coordinators provided instrumental and emotional support to clients, facilitating service linkages and ensuring clients felt supported.

### 4.2 Care Coordination

Participants' statements on care coordination fell into three main categories: the distinction between care coordination and case management, coordination of services, and support for clients and other providers. While care coordination functioned slightly differently at Agency A and Agency B, focus groups from both identified similar benefits of care coordination in the successful integration of services and support for clients and providers. Conversely, the confusion around differences between care coordination and case management was present at both agencies, though conversations at Agency A primarily centered on delineating the roles and responsibilities of care coordinators and case managers, while conversations with Agency B generally involved questioning the fundamental distinctions between these roles.

Participants from both agencies noted that care coordinators efficiently linked clients to services, particularly transportation; addressed crises and emergent situations promptly; worked productively with external organizations to obtain information and resources; and helped clients who might otherwise have fallen through the cracks. Similarly, staff from both agencies discussed the support that care coordination teams provided to clients and other providers. While both agencies acknowledged this support, participants from Agency B reported more varied experiences and perspectives regarding the level of support care coordinators were able to provide. Multiple departments within Agency B provided their own care coordination and worked with special populations that they believed would require care coordinators with specialized training. Participants who worked directly with care coordinators and were most familiar with their work generally reported that collaboration with care coordinators reduced individual provider burden by streamlining service coordination.

While feedback on care coordinators and the support and service coordination they provide was predominantly positive, significant confusion persisted regarding the distinctions between care coordination and case management. Care coordinators from both agencies emphasized the flexibility of their role compared to case managers, noting that freedom from billable hour constraints allowed care coordinators to provide support to clients in urgent situations. This flexibility enabled care coordinators to assume some case management responsibilities during case management interruptions or delays at both agencies.

### 4.3 Communication and Collaboration

Participants' discussions about communication and collaboration were organized into three main categories: challenges in communication across teams, facilitative strategies for improved collaboration, and benefits of technology and physical co‐location. Both agencies reported increased communication and collaboration within the CCBHC, though they also encountered some challenges. For example, the case management team from Agency A reported that they occasionally found the level of contact from care coordinators overwhelming, though they acknowledged it served as a useful reminder of overlooked tasks. They also noted that care coordinators sometimes had different expectations regarding the speed of task completion, posing challenges for case managers handling large caseloads. There was a perception that care coordinators may not fully understand the barriers faced by some case management clients.

Agency B's larger size created unique challenges, particularly around communication across departments. Some teams reported difficulty communicating across departments, while others had limited awareness of care coordinators' roles. Care coordinators proposed organizing meetings to discuss workflows and referral processes, and leadership planned to educate staff about the CCBHC model to address knowledge gaps.

Both agencies implemented strategies to improve collaboration. Agency A utilized an electronic health record system supporting multiple communication methods. Co‐location of behavioral health and primary care staff within the same building also facilitated smoother communication and collaboration. Agency A also scheduled clients with both teams consecutively on the same day, minimizing the need for multiple visits. Both agencies coordinated effectively with external providers and benefited from interdisciplinary huddle meetings addressing client needs. Communication within Agency A improved as team members better understood each other's roles and leveraged their unique strengths.

### 4.4 Client Benefits

Focus group discussions regarding client benefits clustered into three main categories: improved care, reduced hospitalizations and incarcerations, and psychoeducation. Several aspects of improved care were recognized across different focus groups from both agencies, though with varying emphasis. Similarly, both agencies discussed preventing hospitalizations, but participants from Agency A also addressed preventing incarceration. Finally, psychoeducation was almost entirely discussed by Agency A.

Statements on improved care included more training on EBPs, person‐centered treatment, collaborative care, expanded levels of care and services, and streamlined service delivery. Both agencies addressed most aspects of improved care, with different priorities. Agency A's groups focused on providing compassionate, client‐centered care, promoting collaborative care approaches, and streamlining service linkage through care coordinators. Agency B participants centered discussions were on increased EBP training, expanding available care types and services, and utilizing care coordinators to support linkage and reduce wait times for services.

Both Agency A and Agency B discussed preventing hospitalizations in similar terms. The increased quality and quantity of ongoing services available to clients through the CCBHC was identified as a crucial benefit. Ongoing services were viewed as an approach to maintain client stability in the community and prevent escalation to the point of requiring hospitalization. Agency A further extended this benefit to preventing escalations that lead to law enforcement involvement.

Psychoeducation was exclusively discussed by Agency A. Educating clients, families, and the broader community about mental health and wellbeing was identified as a significant client benefit. Participants from Agency A frequently mentioned teaching clients about the connection between physical and mental health, educating families about their loved ones' conditions and supportive techniques, and informing other community organizations, including police and pharmacies, about mental health. Community psychoeducation and collaborations were noted as particularly beneficial, with police officers developing improved understanding of the challenges faced by individuals with severe mental illnesses and available resources.

### 4.5 Organizational Change

Focus group conversations of organizational change were categorized into four topics: CCBHC planning and startup, implementation of programs and services, communication with staff about the CCBHC, and the integration of care. Participants from both agencies identified the CCBHC startup period as an important time to plan CCBHC activities and implementation. Further, both agencies reported that it took the first year of the grant to fully implement the CCBHC and begin to see results. Agency A extended its CCBHC planning phase, and while this reduced overall enrollment initially, participants expressed that the additional planning time ultimately enhanced their organizational change process, allowing them to develop a shared understanding of the CCBHC.

Agency B experienced similar enrollment challenges. Their primary enrollment barrier was the implementation and administration of the National Outcomes Measures (NOMs), which were part of the requirement of the grant. Agency B's community outreach was successful, and clients were provided with services, but focus group participants indicated that the NOMs assessment process was burdensome for many of their clients, impacting data collection rates.

Another significant barrier in implementing programs and services experienced by both agencies was staffing. Agency A and Agency B both encountered difficulties with staff turnover and vacancies, addressing these staffing issues by utilizing existing staff when possible. Despite these challenges, both agencies used CCBHC funding to expand programs and services available for clients, ranging from creating ACT and care coordination programs to expanding existing SUD and youth programs.

The most notable differences in organizational change between the agencies involved staff communication about the CCBHC and care integration. In particular, Agency B's challenges with departmental siloing and communication were reflected during focus groups: staff who did not work directly with care coordinators lacked awareness of said role's purpose or its distinction from case management, and in some cases, staff members were unaware of who the care coordinators were or how to contact them. Agency A staff also experienced some difficulties differentiating between case managers and care coordinators; however, as the grant progressed, this was mitigated through administration and leadership communication and training on the CCBHC. While service providers from Agency B initially expressed confusion, they shared examples of how the CCBHC and care coordinators benefited their clients over time.

Both agencies successfully coordinated care internally and externally and experienced organizational change, but Agency A embraced that change to a greater extent. Agency B leadership noted progress in team communication and streamlined processes, but acknowledged that culture change and full integration were still developing. This was echoed in other Agency B focus groups, where participants who initially questioned the CCBHC's purpose began to recognize its benefits during final focus group sessions. Agency A leadership reported significant progress toward integrated care, with a clear vision supported by an internal group addressing cultural transformation. Agency A demonstrated intentional communication about CCBHC through active staff collaboration, with care coordinators feeling empowered by leadership to advocate for clients. Staff across different levels shared support for CCBHC's underlying concepts and benefits.

### 4.6 Sustainability

Discussions about sustainability focused on three primary areas: concerns about post‐grant funding, efforts to maintain services beyond the grant period, and state‐level advocacy for continued support. The sustainability of care coordination services emerged as a concern for both agencies, particularly highlighted during the focus groups conducted toward the end of the grant period. Agency A emphasized the importance of care coordination for increasing service linkage and reducing ED visits and hospitalizations for clients, stressing the need for the continuity of care coordination services beyond the grant period. Staff hoped for the promotion of care coordination at the state level. Agency B also shared its efforts to ensure the viability of the ACT team beyond the funding period.

## 5 Discussion

The current study explored provider experiences with CCBHC implementation across two agencies that differed in size. Participants included both direct service professionals and leadership/administrative teams, with focus group discussions conducted separately to create an environment where participants could comfortably share their experiences. Qualitative analysis of focus group interviews revealed six key themes: access to services, care coordination, communication and collaboration, client benefits, organizational change, and sustainability.

Providers from both agencies observed benefits such as improved care through EBP training, person‐centered treatment, keeping clients out of jail and hospitals, and improved coordination, findings that align well with the emerging CCBHC literature [4, 8]. However, access to social resources like housing and transportation that often fell out of the scope of agency services remained a persistent challenge throughout the grant period, pointing to a major social infrastructure problem. The housing crisis is disproportionately affecting individuals with low income and those with complex needs. Recent data show that for every 100 extremely low‐income individuals, only 35 affordable rental homes are available, while ~75% of households qualifying for federal housing assistance cannot obtain housing due to insufficient funding [14]. This combination of lack of affordable housing, shortage of public benefits, and financial hardship contributes to homelessness [14]. Similarly, transportation was another significant barrier for CCBHC clients, limiting their ability to access services at the clinics.

Care coordination was perceived as valuable, although some providers at both agencies expressed confusion about the specific role of care coordinators yet still found them complimentary to their roles. Providers frequently emphasized that care coordinators were crucial in supporting both clients and other providers by alleviating workload burdens. Care coordinators were viewed as particularly helpful in connecting clients to services and resources, largely due to their flexibility from not being constrained by billable hours, allowing them to provide additional support when clients needed it the most. A systematic review found that care coordination improved outcomes for clients with severe mental illness and other medical conditions such as congestive heart failure and diabetes mellitus [15]. Many clients with comorbid conditions fall through the cracks when they lack support outside of structured services [16]. Navigating services independently can be daunting for clients, making care coordination a crucial support mechanism.

Both agencies experienced organizational change because of the CCBHC, though some of their strategies differed. One agency intentionally informed all the providers about the CCBHC, whereas the other agency did not disseminate this information. It is worth noting that these agencies differed in size, with the latter being significantly larger, which may have complicated the information dissemination process. Despite these differences, both agencies shared a vision of integrated care as their ultimate goal and observed cultural shifts within their organizations. Sustainability of the CCBHC was a significant concern at the end of the grant period, especially the viability of care coordination. CCBHCs are implemented through Section 223 CCBHC demonstration program, the Substance Abuse and Mental Health Services Administration (SAMHSA) CCBHC expansion grants, and independent state implementation via state Medicaid programs [17]. For these two agencies, which received expansion grants limited to 2 years, developing sustainability plans after the grant period was a critical challenge, as neither operated in a state that participated in the demonstration or had an established state CCBHC program.

## 6 Limitations

This study has a few limitations. Providers were recruited from two agencies operating in a state without a state CCBHC program. This limits the generalizability of the findings, as the experiences of agencies in states with established CCBHC programs may differ from those of providers in the current study. However, the inclusion of two agencies that served different communities and varied in size provides valuable insights into the field.

The focus groups had a wide variety in size because many of the groups were conducted during team meetings, and some of the teams had fewer members, while other teams were larger and integrated staff from other parts of the agency. While the researchers requested that staff members only attend one focus group for each time point, some overlap was unavoidable because groups were primarily conducted during team meetings. This issue illustrates some of the challenges and possible solutions for conducting naturalistic research in busy agency settings.

Another limitation is the absence of input from clients who received CCBHC services, as this was beyond the scope of our study. Client perspectives likely offer valuable insights that may differ from yet complement provider viewpoints. Future research should prioritize gathering these client experiences to develop a more comprehensive understanding of the impact of CCBHCs.

## 7 Implications for Practice, Policy, and Research

Integrated care is critical to optimize the outcomes of individuals with behavioral health problems, especially because they are at increased risk of developing comorbid medical conditions. The CCBHC model represents one approach to delivering integrated care, and it is becoming increasingly available across the United States. Providers in this study shared many positive experiences as well as some challenges as they worked through the implementation of CCBHC.

One of the main issues raised from this study is sustainability. The two agencies in our study were SAMHSA grantees operating in a state without a certification program, exemplifying the sustainability challenges faced by many CCBHCs. These agencies face uncertainty when grants expire, as SAMHSA grant funding is provided for a set period of time. Clinics continue to bill Medicaid and other payers as usual during that period and beyond; however, they are unable to fund care coordinators after the grant ends since they are not billable under the traditional fee‐for‐service payment system alone. In contrast, state CCBHC programs provide prospective payment systems that fund expanded services while offering flexibility that enables clinics to serve emerging needs in their communities and improve their infrastructure [18].

Role confusion between case managers and care coordinators impacts implementation success and staff efficiency. Clearer role definitions could enhance workflow efficiency. SAMHSA's CCBHC certification standards [5] require CCBHCs to directly provide care coordination, while targeted case management can be delivered either by the CCBHC or through DCOs. While care coordination serves all CCBHC clients, targeted case management offers more intensive services for clients with complex needs. Although this distinction offers some guidance for role differentiation, our findings reveal it proves insufficient in practice. This gap indicates a need for more detailed implementation guidance at state and national levels.

Another significant challenge involved connecting clients to scarce community social resources such as housing and transportation. Since CCBHCs serve clients with complex needs who often experience homelessness and transportation barriers, it becomes evident that these centers cannot fulfill their mission of whole‐person care without addressing these essential needs. Thus, strengthening the social infrastructure of communities is essential for CCBHCs to comprehensively meet client needs and maximize the effectiveness of clinical interventions. While the CCBHC model shows promise for delivering integrated, comprehensive care, further rigorous studies are needed to evaluate its effectiveness in improving overall health outcomes and client wellbeing.

## Conflicts of Interest

The authors declare no conflicts of interest.

## Data Availability

Research data are not shared.

## References

[1] B. G. Druss and R. A. Rosenheck , “Mental Disorders and Access to Medical Care in the United States,” American Journal of Psychiatry 155, no. 12 (1998): 1775–1777, 10.1176/ajp.155.12.1775. 9842793

[2] Z. Fan , Y. Wu , J. Shen , T. Ji , and R. Zhan , “Schizophrenia and the Risk of Cardiovascular Diseases: A Meta‐Analysis of Thirteen Cohort Studies,” Journal of Psychiatric Research 47, no. 11 (2013): 1549–1556, 10.1016/j.jpsychires.2013.07.011. 23953755

[3] M. T. Schulte and Y. I. Hser , “Substance Use and Associated Health Conditions Throughout the Lifespan,” Public Health Reviews 35, no. 2 (2014): 1–27, 10.1007/BF03391702. 28366975 PMC5373082

[4] U.S. Department of Health and Human Services , “Implementation Findings From the National Evaluation of the Certified Community Behavioral Health Clinic Demonstration,” 2020, https://aspe.hhs.gov/sites/default/files/private/pdf/263986/CCBHCImpFind.pdf.

[5] Substance Abuse and Mental Health Services Administration , “Certified Community Behavioral Health Center (CCBHC) Certification Criteria,” 2023, https://www.samhsa.gov/sites/default/files/ccbhc‐criteria‐2023.pdf.

[6] Y. Hu , V. Stanhope , E. B. Matthew , and D. M. Baslock , “A Brief Report on Certified Community Behavioral Health Clinics Demonstration Program,” Social Work in Mental Health 19, no. 6 (2021): 534–541, 10.1080/15332985.2021.1929663.

[7] National Council for Mental Wellbeing , “CCBHC Impact Report,” 2024, https://www.thenationalcouncil.org/resources/2024‐ccbhc‐impact‐report/.

[8] National Council for Mental Wellbeing , “Transforming State Behavioral Health Systems: Findings From States on the Impact of CCBHC Implementation,” 2022, https://www.thenationalcouncil.org/wp‐content/uploads/2021/10/21.10.04_CCBHC‐State‐Impact‐Report.pdf?daf=375ateTbd56.

[9] National Council for Mental Wellbeing , “Leading a Bold Shift in Mental Health and Substance Use Care: CCBHC Impact Report,” 2021, https://www.thenationalcouncil.org/wp‐content/uploads/2022/09/052421_CCBHC_ImpactReport_2021_Final.pdf.

[10] J. D. Brown , K. A. Stewart , R. L. Miller , et al., “Impacts of the Certified Community Behavioral Health Clinic Demonstration on Emergency Department Visits and Hospitalizations,” Psychiatric Services 74, no. 9 (2023): 911–920, 10.1176/appi.ps.20220410. 36916061

[11] D. Maeng , P. Walsh , G. Nasra , and H. B. Lee , “Certified Community Behavioral Health Clinic Demonstration Impact on Health Care Utilization Among Non‐Medicaid Patients With Severe Mental Illnesses,” Population Health Management 27, no. 5 (2024): 345–352, 10.1089/pop.2024.0103. 39133113

[12] A. Burner , C. Wahl , and L. Struwe , “Factors to Improve Reverse Integration: A Mixed Method Embedded Design Study,” Community Mental Health Journal 60, no. 3 (2024): 525–535, 10.1007/s10597-023-01203-0. 37985631

[13] K. Malterud , “Systematic Text Condensation: A Strategy for Qualitative Analysis,” Scandinavian Journal of Public Health 40 (2012): 795–805, 10.1177/1403494812465030. 23221918

[14] National Alliance to End Homelessness , “The Affordable Housing Gap: A Crisis for Low‐Income Renters,” 2025, https://endhomelessness.org/a‐shortage‐of‐affordable‐housing/.

[15] K. M. McDonald , V. Sundaram , D. M. Bravata , et al., Closing the Quality Gap: A Critical Analysis of Quality Improvement Strategies (Vol. 7: Care Coordination) (Agency for Healthcare Research and Quality (US), 2007). 20734531

[16] Institute of Medicine (US) Committee on Crossing the Quality Chasm: Adaptation to Mental Health and Addictive Disorders , Improving the Quality of Health Care for Mental and Substance‐Use Conditions: Quality Chasm Series (National Academies Press (US), 2006). 20669433

[17] Substance Abuse and Mental Health Services Administration , “Certified Community Behavioral Health Clinics,” https://www.samhsa.gov/communities/certified‐community‐behavioral‐health‐clinics/section‐223.

[18] National Council for Mental Wellbeing , “CCBHCs: A Vision for the Future of Community Behavioral Health Care,” 2023, https://www.thenationalcouncil.org/resources/ccbhcs‐a‐vision‐for‐the‐future/.

